# Does Cognitive Broadening Reduce Anger?

**DOI:** 10.3389/fpsyg.2018.02665

**Published:** 2019-01-08

**Authors:** Elizabeth Summerell, Cindy Harmon-Jones, Nicholas J. Kelley, Carly K. Peterson, Klimentina Krstanoska-Blazeska, Eddie Harmon-Jones

**Affiliations:** ^1^School of Psychology, The University of New South Wales, Sydney, NSW, Australia; ^2^Department of Psychology, Northwestern University, Evanston, IL, United States; ^3^Minneapolis Veterans Affairs Health Care System, Minneapolis, MN, United States

**Keywords:** cognitive scope, anger, emotion, motivation, replication

## Abstract

Past theory and research have suggested that motivationally intense affective states narrow cognitive scope. Research has also suggested manipulations that broaden cognitive scope reduce responses to appetitive positive affective stimuli and disgusting stimuli, thus suggesting that cognitive broadening reduces motivational intensity. This led to the hypothesis that cognitive broadening would reduce the approach-motivated negative emotion of anger. Seven studies assessed the effect of cognitive broadening on reported trait anger, state anger, attitudes toward anger, attributions of anger to ambiguous pictures, and accessibility of aggressive words. Results from individual studies found mixed support for these predictions. A meta-analysis, however, suggested a small but significant effect on trait anger/aggression and attitudes toward anger across studies. These results may indicate that cognitive scope, as manipulated in these studies, has a small effect on anger-related responses. Discussion speculates on potential explanations of these findings, and their importance for informing future research.

## Introduction

The scientific process should be cumulative and self-correcting. In reality, publication bias and the tendency to retire null results to the file drawer often prevents this goal from being accomplished. This retirement may also mislead as accumulating effects over several replications may yield evidence of an effect in a meta-analysis, even though not all of the experiments attain conventional statistical significance when viewed alone (Braver et al., 2014). In this article, we present several studies that were designed to test the prediction that increasing the breadth of cognitive scope would reduce anger-related responses, along with a meta-analysis to assess the strength of the effect overall. As we elaborate below, this prediction was based on a programmatic body of research wherein we hypothesized in the discussion section of a previous paper that *“a manipulated broadening of cognitive scope would influence responses to anger-inducing stimuli”* (Gable et al., 2015).

Cognitive scope refers to the broadening or narrowing of cognitive processes (Harmon-Jones et al., 2012). It has featured widely in discussions on the influence of emotion on cognition (e.g., Fredrickson, 2001). Cognitive scope has been operationalized at perceptual, attentional, and conceptual levels, and measured using a variety of tasks assessing breadth of attention and perception, unusualness of word associations, and cognitive categorization (Gable and Harmon-Jones, 2010b; Harmon-Jones et al., 2012; Kaplan et al., 2012; Burgoon et al., 2013). Motivational intensity is defined as the strength of the urge to approach or avoid, and can range from low to high (Gable and Harmon-Jones, 2010b; Harmon-Jones et al., 2013b; Harmon-Jones and Gable, 2018). Motivational intensity is distinct from arousal. Motivational intensity involves an impetus to act, whereas arousal does not necessarily (Gable and Harmon-Jones, 2013).

Past research has found that motivationally intense affects are associated with a narrowing of cognitive processes (Gable and Harmon-Jones, 2010a, 2011a; for review, see Gable et al., 2015). Other studies have found that broadening cognitive processes reduces emotive responses to motivationally intense affect (Gable and Harmon-Jones, 2011b, 2012). The present research aimed to explore this potential bidirectional relationship between motivational intensity and cognitive scope, focusing on anger, a motivationally intense negative affect.

The motivational dimensional model of affect (Gable and Harmon-Jones, 2010b) proposes that motivational intensity is the underlying mechanism for the influence of affective states on cognitive scope. Motivationally intense affects cause a narrowing of cognitive processes (Gable and Harmon-Jones, 2010a, 2011a; Gable et al., 2015). Empirical results have revealed that attentional narrowing occurs following inductions of affective states high in motivational intensity, irrespective of valence (Gable and Harmon-Jones, 2008, 2010a, 2011a; Harmon-Jones and Gable, 2009; Hicks et al., 2012; Kaplan et al., 2012; Gable et al., 2015). For example, individuals display less inclusive (more narrow) categorizations following the manipulation of high-approach positive affect compared to low-approach positive affect (Price and Harmon-Jones, 2010; Gable and Harmon-Jones, 2011a). Individuals also display less inclusive categorizations following the induction of anger (Gable et al., 2015). Conversely, affective states low in motivational intensity broaden cognitive scope. For example, participants have faster reaction times to global compared to local targets on a global-local letter task after viewing sad images (Gable and Harmon-Jones, 2010a).

Other studies found that broadening cognitive processes reduces motivationally intense affective responses (Gable and Harmon-Jones, 2011b, 2012). In one study, a manipulated increase in global attention led to a reduction in early neural responses indexing attention capture (i.e., the N1 event-related potential component) when viewing appetitive pictures (Gable and Harmon-Jones, 2011b). That is, compared to a manipulated increase in local attentional scope, a manipulated increase in global attentional scope caused smaller N1 amplitudes toward pictures that evoked desire. Similarly, a manipulated increase in global attention led to a reduction in neural responses when viewing aversive pictures (Gable and Harmon-Jones, 2012). That is, compared to a manipulated local attentional scope, a manipulated global attentional scope caused smaller N1 amplitudes toward pictures that evoked disgust. Together, the reviewed research suggests a bidirectional relationship between motivational intensity and cognitive scope, such that increased motivation narrows cognitive scope, and broadened cognitive scope reduces motivational intensity.

Evidence has suggested that processing in a cognitively broad manner (i.e., processing global features) is associated with activity in the right cerebral hemisphere, whereas processing in a cognitively narrow manner (i.e., processing local features) is associated with activity in the left cerebral hemisphere (Volberg and Hübner, 2004; Volberg et al., 2009; Boksem et al., 2012). For example, research has found that attending to global features causes greater relative right hemispheric activation (Fink et al., 1996, 1997), and activation of the right hemisphere has been found to enhance processing of global stimuli (Gable et al., 2013). Other research has suggested that the hemispheric asymmetries associated with cognitive scope (global/local processing) may be specific to the inferior parietal lobe/superior temporal gyrus (Weissman and Woldorff, 2004).

The broadening of cognitive scope likely confers several adaptive advantages to our cognitive system. For example, Navon (1977, p. 381) proposed that it, “… has a number of possible advantages such as utilization of low-resolution information, economy of processing resources, and disambiguation of indistinct details.” Other advantages likely associated with the broadening of cognitive scope include: (1) increased processing of peripheral information (Carver, 2003; Gable and Harmon-Jones, 2010b); (2) assisting with the processing of past events and consideration of new goals and opportunities (Kaplan et al., 2012); and (3) facilitating conservation of energy and resources following successful goal pursuit and the reallocation of resources to distal stimuli (Carver, 2003; Harmon-Jones et al., 2013a). Of course, the narrowing of cognitive scope also likely confers several adaptive advantages as well, primarily linked to focusing attention and other resources on the acquisition of desired goals (Gable and Harmon-Jones, 2008, 2010a,b).

The physiological correlates and possible adaptive functions of a broadened cognitive scope are consistent with the prediction that a broadened cognitive scope will reduce anger-related responses. That is, a broadened cognitive scope is associated with greater relative right hemispheric processing, whereas anger is associated with greater relative left frontal hemispheric processing (see review by Harmon-Jones and Gable, 2018). Perhaps the greater relative right hemispheric processing associated with a broadened cognitive scope reduces anger-related responses by decreasing the relative left frontal cortical activity associated with anger, via contralateral inhibition (Schutter and Harmon-Jones, 2013). In addition, if a broadened cognitive scope is associated with increased processing of peripheral information, consideration of new goals and opportunities, and the reallocation of resources to distal stimuli, then these broad adaptive functions seem antithetical to the experience of anger, as anger often involves a focus on a central goal object, consideration of the presently-active goal, and allocation of resources to immediate stimuli.

The present research was designed to test the novel prediction that a broadened cognitive scope would reduce anger-related responses. As reviewed above, only two previous studies have tested whether a manipulated cognitive scope influences emotive responses. These studies examined emotive responses associated with desire and disgust, and they examined early neural responses to pictures. The present research extends this past research by examining anger-related responses and by examining multiple measures of emotive responses.

Cognitive scope was also manipulated in ways that differed from past research. Studies 1–5 manipulated cognitive scope using a sentence unscrambling task. This task was created to closely resemble measures of cognitive categorization used in past studies (e.g., Isen and Daubman, 1984; Price and Harmon-Jones, 2010). To extend the generalizability of the manipulation of cognitive scope, Studies 6 and 7 used more conceptual manipulations of cognitive scope.

Anger was assessed in several ways, which were different than the ones examined in previous studies on the effect of cognitive scope on emotive responses. Studies 1, 2, and 3 examined reported trait anger, state anger, and attitudes toward anger, Studies 4 and 5 examined attitudes toward anger and attributions of anger to ambiguous picture stimuli, Study 6 examined the accessibility of aggression-related words and aggressive motivation, and Study 7 examined the accessibility of aggression-related words and state anger.

We aimed to examine the effect of cognitive scope on several anger-related responses, to increase the generalizability of the tests and results. We predicted that the manipulation of cognitive scope would influence a wide range of anger-related measures. By demonstrating that the cognitive scope manipulation influenced a wide range of anger-related measures, we would be more able to conclude that cognitive scope influenced the psychological response of anger rather than just one measure. Thus, we included assessments of self-reported state and trait anger as well as self-reports of attitudes toward anger (e.g., “I like how it feels when I am furious.” Harmon-Jones, 2004). We also included more implicit measures of anger, such as attributions of anger to ambiguous pictures in which one person may have been displaying anger and the accessibility of aggression-related words (i. e., completion of word fragments).

## Study 1

### Methods

#### Participants

Participants (*n* = 111) were undergraduate students at Texas A&M University who participated in exchange for partial course credit. Other demographic information was not collected. Four participants were excluded due to missing data, leaving 107 participants for analysis. Following the recommendations of others (Simmons et al., 2011) who have suggested that when power analyses are not conducted in advance, we report our data stopping rule: we tested as many participants as possible during the semester and ended data collection when the participant pool closed. Participants provided written informed consent.

#### Materials and Procedure

Participants gave informed consent before being randomly assigned to listen to either an insulting or neutral radio broadcast (from Harmon-Jones et al., 2009). They were then randomly assigned to complete a sentence unscrambling task designed to either narrow or broaden their cognitive scope. This task was created to be similar to measures of cognitive categorization used in past research (e.g., Isen and Daubman, 1984; Price and Harmon-Jones, 2010). Participants were instructed to form 27 grammatically-correct four-word sentences from 27 sets of five words. The task was designed so that all completed sentences were statements sorting an exemplar into one of nine categories: furniture, fruit, vehicles, vegetables, tools, birds, sports, toys, and clothing. There were three sentences for each of the nine categories. An example was provided, so that “are oaks the trees” became “the oaks are trees,” where “oak” is the exemplar and “tree” is the category. Two versions of this task were created by manipulating the strength of the exemplar as a member of the category. Strong exemplars were used to narrow cognitive scope, whereas weak exemplars were used to broaden cognitive scope. The strength of the exemplar was determined based on norms established by Rosch (1975), so that strong exemplars, such as “chair” for furniture or “bananas” for fruit, were ranked among the top three category members, and weak exemplars, such as “telephone” for furniture or “olives” for fruit, were ranked among the bottom three category members.

Following this, participants completed the Expanded Form of the Positive and Negative Affect Schedule (PANAS-X; Watson and Clark, 1994), Attitudes toward Emotions Questionnaire (ATE; Harmon-Jones et al., 2011), and Aggression Questionnaire (AQ; Buss and Perry, 1992). Although the entire PANAS-X and Attitudes toward Emotions questionnaires were administered, only the Anger subscales were analyzed in this and subsequent studies. PANAS-X Anger items were “angry,” “hostile,” “irritable,” “scornful,” and “loathing.” We did not include the item “disgusting” in this anger subscale as has been done in the past, as it is conceptually distinct from anger. For details of additional measures administered but not analyzed or reported in this manuscript see Supplementary Material.

### Results and Discussion

The insulting broadcast evoked significantly more PANAS Anger (*M =* 2.42, *SD* = 0.78, 95% CI [2.20, 2.64]) than the neutral broadcast (*M* = 1.67, *SD* = 0.62, 95% CI [1.51, 1.84]), *F*(1,103) = 29.59, *p* < 0.001, $\eta_{p}^{2}$ = 0.223. Self-reported anger was not influenced by the cognitive scope manipulation (see Table 1), or the broadcast × cognitive scope interaction *F*(1,103) = 0.001, *p* = 0.972. Due to experimenter error, data from the sentence unscrambling task could not be matched to outcome data. Consequently, exclusions could not be made based on manipulation check failure.

**Table 1:** Effect of cognitive scope condition on attitude toward anger and trait aggression subscales – Study 1.

	Narrow (*n* = 54)		Broad (*n* = 53)				
	*M* (*SD*)	95% CI	*M* (*SD*)	95% CI	*F*	*p*	$\eta_{p}^{2}$
ATA	1.76 (0.64)	[1.59, 1.94]	1.57 (0.54)	[1.42, 1.72]	3.07	0.083	0.029
Trait Anger	3.06 (0.64)	[2.88, 3.23]	2.92 (0.73)	[2.72, 3.12]	1.18	0.279	0.011
Trait Hostility	2.59 (0.83)	[2.37, 2.82]	2.12 (0.84)	[1.89, 2.35]	8.90	0.004	0.080
Trait Phys. Aggr.	2.64 (1.13)	[2.33, 2.95]	2.42 (0.70)	[2.22, 2.61]	1.48	0.227	0.014
Trait Verb. Aggr.	3.22 (1.12)	[2.91, 3.52]	3.06 (0.89)	[2.82, 3.31]	0.69	0.410	0.007
PANAS Anger	1.67 (0.62)	[1.51, 1.84]	2.42 (0.78)	[2.20, 2.64]	0.19	0.666	0.002

*ATA, Attitude toward Anger; Trait Phys. Aggr., Trait Physical Aggression; Trait Verbal Aggr., Trait Verbal Aggression.*

Cognitive scope exerted a marginally significant effect on attitudes toward anger (see Table 1), such that participants in the broad condition liked anger less than those in the narrow condition. Broadcast type exerted no main effect (see Table 1), but a marginally significant broadcast × cognitive scope interaction occurred, *F*(1,103) = 3.76, *p* = 0.06, $\eta_{p}^{2}$ = 0.035. When the radio broadcast was followed by the *cognitive narrowing condition*, participants who listened to the insulting broadcast reported *more favorable* attitudes toward anger (*M* = 1.93, *SD* = 0.64, 95% CI [1.67, 2.19]) than those who listened to the neutral broadcast (*M* = 1.61, *SD* = 0.61, 95% CI [1.37, 1.84]), *t*(52) = 1.91, *p* = 0.06, Cohen’s *d* = 0.519. Within the *cognitive broadening condition*, attitudes toward anger did not differ between the insulting (*M* = 1.51, *SD* = 0.50) and neutral (*M* = 1.63, *SD* = 0.59) broadcast conditions *t*(51) = 0.78, *p* = 0.441.

When examining the trait aggression subscales separately, the cognitive scope manipulation had a significant effect on trait hostility (see Table 1). Participants in the cognitive narrowing condition reported more trait hostility than those in the cognitive broadening condition. Trait hostility was not influenced by broadcast type or the broadcast × cognitive scope interaction. No statistically significant main effects or interactions were observed for physical aggression, verbal aggression, or anger, however the means on all subscales of the aggression questionnaire were higher in the narrow condition as predicted (see Table 1).

These results suggest that broadening cognitive scope caused reduced self-reported trait hostility and less favorable attitudes toward anger. We assume that the trait aggression scale was influenced by the manipulation because it acted as a proxy for state anger. Traits are conceptualized as enduring but abstract constructs that represent a proneness to act in a particular way, and are not typically associated with discrete, subjective feelings (Fridhandler, 1986). However, trait self-report measures only serve as a proxy for actual traits, rather than as direct measures of the traits themselves, and thus responses on trait measures may be affected by the situation. Indeed, research suggests that the measurement of affective traits can be influenced by situational variables such as mood (Schwarz and Clore, 1983; Brose et al., 2013).

In addition, perhaps the state anger manipulation overwhelmed the cognitive scope manipulation, which prevented the cognitive scope manipulation from influencing state anger to the broadcast. In other words, the state anger manipulation may have been of much higher intensity than the cognitive scope manipulation. Therefore, in Study 2, we removed the state anger manipulation and only tested whether the effect of cognitive scope on trait anger-related responses would replicate.

## Study 2

To test if the results for Study 1 were replicable, Study 2 examined whether broadening, as compared to narrowing, cognitive scope would decrease trait anger and attitudes toward anger, in the absence of an anger manipulation.

### Methods

#### Participants

Participants (*n* = 64) were undergraduate students at Texas A&M University who participated in exchange for partial course credit. Twelve participants were excluded for failing to correctly complete 75% or more of the sentences correctly, leaving 52 participants for analysis (26 females). The final sample had a mean age of 18.67 (*SD* = 0.94), and reported the following ethnicities: White (78.85%), Hispanic (9.62%), Black (1.92%), Asian (5.77%), Sri Lankan/Caucasian (1.92%), and Indian (1.92%). Following the recommendations of others (Simmons et al., 2011) who have suggested that when power analyses are not conducted in advance, we report our data stopping rule: we tested as many participants as possible during the semester and ended data collection when the participant pool closed. Participants provided written informed consent.

#### Materials and Procedure

Participants were randomly assigned to complete the narrow or broad sentence unscrambling task used in Study 1. They then completed the ATE (Harmon-Jones et al., 2011) and the Aggression Questionnaire (Buss and Perry, 1992).

### Results

Because this study was designed to replicate the previous one, the predictions were directional, derived from theory, and specified in advance. Thus, they were evaluated using a one-tailed criterion of significance (Rosenthal et al., 2000). All subsequent studies were also analyzed in this way.

Compared to participants in the narrow condition, those in the broad condition reported less favorable attitudes toward anger, lower trait anger, and lower trait physical aggression (Table 2), conceptually replicating Study 1. Although the mean for trait verbal aggression and trait hostility were lower in the broad condition as predicted, the differences were not statistically significant.

**Table 2:** Effect of cognitive scope condition on attitudes toward anger and trait aggression subscales – Study 2.

	Narrow (*n* = 24)		Broad (*n* = 28)				
	*M* (*SD*)	95% CI	*M* (*SD*)	*95% CI*	*t*	*p*	*d*
ATA	2.14 (0.84)	[1.78, 2.50]	1.67 (0.69)	[1.41, 1.94]	2.19	0.017	0.611
Trait Anger	2.49 (0.87)	[2.13, 2.86]	1.92 (0.61)	[1.69, 2.16]	2.77	0.004	0.759
Trait Hostility	2.48 (0.92)	[2.09, 2.87]	2.29 (0.62)	[2.05, 2.53]	0.90	0.187	0.246
Trait Phys. Aggr.	2.51 (0.99)	[2.09, 2.93]	2.06 (0.77)	[1.77, 2.36]	1.83	0.037	0.503
Trait Verb. Aggr.	3.09 (0.77)	[2.77, 3.42]	2.83 (0.79)	[2.52, 3.13]	1.21	0.116	0.337

*For ATA, *n* = 23 in Narrow condition due to missing data. Trait Phys. Aggr., Trait Physical Aggression; Trait Verb. Aggr., Trait Verbal Aggression; ATA, Attitude toward Anger.*

Study 2 partially replicated the results from Study 1. As with Study 1, we assume that the trait aggression scale and attitudes toward anger were influenced by the manipulation because they acted as proxies for state anger.

## Study 3

Study 3 was conducted to attempt to replicate Study 2 using an online sample of participants. We conducted this replication to test whether the effects obtained in Studies 1 and 2 would generalize to a different sample of participants. In Studies 1 and 2, the participants were students from a public university in the United States of America (USA). In Study 3, participants were individuals from the USA recruited using Amazon’s Mechanical Turk (MTurk); they were older than the samples from Studies 1 and 2.

### Methods

The methods used in Study 3 were identical to those used in Study 2.

#### Participants

An *a priori* power analysis indicated that a sample of 102 participants was required to detect an effect size of *d* = 0.50 with 80% power, using a one-tailed criterion of significance. As online studies often lose participants who fail to follow instructions, we doubled this sample size. Participants (*n* = 206) were US residents recruited using Amazon’s MTurk who participated in exchange for $2.50. Several participants (*n* = 119) were excluded for unsatisfactorily completing the cognitive scope manipulation, leaving 86 participants for analysis (39 female). The final sample had a mean age of 37.00 (*SD* = 10.81), and reported the following ethnicities: European/White (76.74%), African/Black (3.49%), Asian (8.14%), Hispanic/Latino (8.14%), Native American (1.16%), and Other (2.32%). Participants provided informed consent by entering their initials into a textbox.

#### Materials and Procedure

Participants were randomly assigned to condition to complete the cognitive scope manipulation used in Studies 1 and 2. Following this, participants completed the Aggression Questionnaire and the ATE.

### Results and Discussion

Participants in the broad condition did not differ significantly from those in the narrow condition on the dependent variables (Table 3).

**Table 3:** Effect of cognitive scope condition on attitudes toward anger and trait aggression subscales – Study 3.

	Narrow (*n* = 43)		Broad (*n* = 43)				
	*M* (*SD*)	95% CI	*M* (*SD*)	95% CI	*t*	*p*	*d*
ATA	1.91 (0.52)	[1.75, 2.07]	1.82 (0.43)	[1.69, 1.96]	0.86	0.196	0.185
Trait Anger	1.77 (0.76)	[1.54, 2.01]	1.89 (0.85)	[1.63, 2.16]	–0.69	*ns*	0.149
Trait Hostility	2.10 (0.97)	[1.81, 2,40]	2.15 (0.80)	[1.91, 2.40]	–0.26	*ns*	0.056
Trait Phys. Aggr.	1.84 (0.61)	[1.65, 2.03]	1.88 (0.70)	[1.67, 2.10]	–0.31	*ns*	0.067
Trait Verb. Aggr.	2.37 (0.70)	[2.15, 2.58]	2.41 (0.81)	[2.16, 2.66]	–0.28	*ns*	0.061

*Trait Phys. Aggr., Trait Physical Aggression; Trait Verbal Aggr., Trait Verbal Aggression; ATA, Attitude toward Anger. Because one-tailed *t*-tests were conducted, where means indicate that results were not in the predicted direction, *p*-values are reported as non-significant, abbreviated *ns*.*

Study 3 did not replicate the results of Study 2, which found reductions in trait aggression measures following cognitive broadening. However, this result may not be a reliable indicator of the presence or absence of an effect, due to the large number of exclusions made because of poor performance on the cognitive scope manipulation. A fourth study was conducted to test the hypothesis using different dependent measures.

## Study 4

Study 4 examined whether broadening cognitive scope would decrease angry interpretations of ambiguous stimuli and attitudes toward anger. In this study, we used a more subtle induction of anger to better match the strength and intensity of the cognitive scope manipulation, because of our concern that the state anger induction used in Study 1 was much more intense than the broadened cognitive scope induction.

### Methods

#### Participants

Undergraduate psychology students (*n* = 133) from the University of New South Wales participated in exchange for partial course credit. Several participants (*n* = 70) were excluded for unsatisfactorily completing the cognitive scope manipulation, leaving 63 participants for analysis (43 female). The final sample had a mean age of 19.03 (*SD* = 1.83), and reported the following ethnicities: European/White (39.68%), Asian (46.03%), Hispanic/Latino (1.59%), and Other (12.70%). Following the recommendations of others (Simmons et al., 2011) who have suggested that when power analyses are not conducted in advance, we report our data stopping rule: we tested until all course credit allocated for the study was exhausted. Participants provided written informed consent.

#### Materials and Procedure

Participants provided informed consent and demographic information before completing a practice trial of the task created for this study. This task required participants to rate simple line drawings of individuals engaging in activities according to how much they perceive the individual depicted wants to make someone else feel bad (anger), wants to reach a goal (determination), or enjoys the moment (enjoyment). The drawings were adapted from the Operant Motive Test (Kuhl and Scheffer, 2002). Only anger scenes are reported in the manuscript (for details of determination and enjoyment scenes see Supplementary Material). Participants were then randomly assigned to complete the broad or narrow cognitive scope manipulation used in Studies 1–3. Following this, participants completed the task described above and the ATE (Harmon-Jones et al., 2011).

### Results

Participants in the broad condition reported marginally significantly less favorable attitudes toward anger. The means for anger scenes did not significantly differ between conditions, although the means were lower in the broad condition as expected (Table 4).

**Table 4:** Effect of cognitive scope condition on attitudes toward anger and ratings of anger scenes – Study 4.

	Narrow (*n* = 28)		Broad (*n* = 35)				
	*M* (*SD*)	95% CI	*M* (*SD*)	95% CI	*t*	*p*	*d*
ATA	2.07 (0.58)	[1.84, 2.30]	1.91 (0.33)	[1.79, 2.02]	1.39	0.085	0.342
Scenes Anger	6.39 (1.30)	[5.89, 6.90]	6.03 (1.22)	[5.61, 6.45]	1.14	0.129	0.288

*ATA, Attitude toward Anger.*

Because a high percentage of participants had difficulty following the cognitive scope instructions, a fifth study, sampling only participants for whom English was their first language, was next conducted.

## Study 5

Study 5 was identical to Study 4, but sampled participants for whom English was their first language. This was done in an attempt to remedy the problem in Study 4 of so many participants unsatisfactorily completing the cognitive scope manipulation. We speculated that because the cognitive scope manipulation is linguistically complex, more English language fluency may be needed to successfully complete it.

### Methods

#### Participants

An *a priori* power analysis indicated that a sample of 102 participants was required to detect an effect size of *d* = 0.50 with 80% power, using a one-tailed criterion of significance. Participants (*n* = 139) were individuals for whom English was their first language, recruited from the undergraduate psychology student pool at the University of New South Wales, or the local community, who participated in exchange for partial course credit or $15 per hour, respectively. Several participants (*n* = 23) were excluded for unsatisfactorily completing the cognitive scope manipulation, leaving 116 participants for analysis (79 females). The final sample had a mean age of 20.38 (*SD* = 4.31), and reported the following ethnicities: European/White (40.52%), Asian (47.41%), African/Black (0.86%), and Other (11.21%). Participants provided written informed consent.

#### Materials and Procedure

Due to large numbers of participants incorrectly completing the cognitive scope manipulation, a short online pre-screening questionnaire was introduced after 60 participants had completed the study. Participants were required to correctly complete six practice items from the cognitive scope manipulation to be eligible to participate. Other than the addition of the pre-screen, Study 5 followed the same procedure as Study 4.

### Results and Discussion

No significant effect of the cognitive scope conditions was observed for attitudes toward anger scores; mean differences were not in the predicted direction. Participants in the broad condition also did not differ from those in the narrow condition on ratings of anger scenes (Table 5).

**Table 5:** Effect of cognitive scope condition on attitudes toward anger and ratings of anger scenes – Study 5.

	Narrow (*n* = 63)		Broad (*n* = 53)				
	*M* (*SD*)	95% CI	*M* (*SD*)	95% CI	*t*	*p*	*d*
ATA	1.54 (0.51)	[1.41, 1.67]	1.63 (0.57)	[1.47, 1.79]	–0.86	*ns*	0.160
Scenes Anger	5.99 (1.56)	[5.59, 6.38]	6.13 (1.51)	[5.71, 6.54]	–0.48	*ns*	0.091

*ATA, Attitude toward Anger. Because one-tailed *t*-tests were conducted, where means indicate that results were not in the predicted direction, *p*-values are reported as non-significant, abbreviated *ns*.*

Overall, the results of Study 5 did not support the hypothesis that broadening cognitive scope would reduce anger responses. Further, the results were not supportive of hypotheses, despite the fact that only participants for whom English was their first language were included.

## Study 6

Study 6 tested the prediction that thinking more broadly, compared to narrowly, about an angering event would reduce state anger. To increase the generalizability of both the cognitive scope manipulation and the assessments of anger, the manipulations and measurements were different from those used in previous studies. The cognitive scope manipulation directed participants to think broadly or narrowly about the anger-inducing event. The assessments of anger were an implicit measure of aggression and self-report items assessing angry motivation.

### Methods

#### Participants

An *a priori* power analysis indicated that a sample of 88 participants was required to detect an effect size of *d* = 0.50 with 75% power, using a one-tailed criterion of significance. Because of the possibility of losing data from online participants, 102 US residents were recruited using Amazon’s Mechanical Turk (MTurk) who participated in exchange for $2.50. Four participants were excluded from analysis; three for not responding to a large proportion of questions, and one for not completing the anger induction, leaving 98 participants for analysis (36 females, 1 other). The final sample had a mean age of 35.47 (*SD* = 11.38), and reported the following ethnicities: White (67.35%), Hispanic/Latino (7.14%), African/Black (9.18%), Asian (13.27%), Native American (1.02%), and Other (2.04%). Participants provided informed consent by entering their initials into a textbox.

#### Materials and Procedure

After consenting to participate, participants completed the Discrete Emotions Questionnaire (DEQ; Harmon-Jones et al., 2016) before writing for 4 min about a person or event that made them angry. Following this, participants were randomly assigned to complete either a broad or narrow cognitive scope task in which they were instructed to provide a reason why they believe the person in the anger scenario behaved the way they did. In the narrow condition, participants were then instructed to elaborate on a single reason for the person’s behavior (i.e., “Please think of three other details that support your original explanation as much as possible”). In the broad condition, participants were instructed to elaborate on different reasons for the person’s behavior (i.e., “Please think of three other explanations that are as different from each as possible”). Many participants did not provide four reasons that were consistent with their assigned condition. To increase the sample size available for analyses, participants were included if they could provide at least two responses consistent with their condition.

Participants then completed the Anderson Word Completion Task (AWCT; Anderson et al., 2003), an implicit measure of aggression, and self-report items assessing angry motivation (i.e., rated the extent to which they wanted to “get revenge,” “tell off,” and “confront” the person who had angered them on a scale ranging from 1 = not at all to 7 = an extreme amount). Finally, they completed the DEQ to assess their emotions during the anger induction.

### Results and Discussion

Self-reported anger significantly increased from baseline in both the broad and narrow conditions after completing the anger task [*t*(102) = 14.18, *p* ≤ 0.001; *t*(90) = 10.71, *p* < 0.001, respectively]. Cognitive scope conditions did not differ significantly on angry/aggressive word accessibility, angry motivation, or DEQ Anger (Table 6).

**Table 6:** Effect of cognitive scope condition on angry word accessibility, angry motivation, and state anger – Study 6.

	Narrow (*n* = 46)		Broad (*n* = 52)				
	*M* (*SD*)	95% CI	*M* (*SD*)	95% CI	*t*	*p*	*d*
AWCT	4.09 (1.96)	[3.50, 4.67]	4.29 (2.23)	[3.67, 4.91]	–0.47	*ns*	0.096
Angry motivation	3.92 (1.95)	[3.34, 4.50]	4.01 (1.77)	[3.52, 4.51]	–0.25	*ns*	0.050
DEQ Anger	4.55 (1.64)	[4.45, 5.24]	4.85 (1.41)	[4.06, 5.04]	0.96	*ns*	0.194

*AWCT, Sum of number of aggressive words on Anderson Word Completion Task. Because one-tailed *t*-tests were conducted, where means indicate that results were not in the predicted direction, *p*-values are reported as non-significant, abbreviated *ns*.*

Results from Study 6 did not support the prediction that cognitive broadening would reduce anger responses. Mean differences between conditions were small and in the opposite direction from predictions.

## Study 7

Study 7 attempted to replicate Study 6 using a different manipulation of cognitive scope. In Study 6 participants were asked to think broadly or narrowly about an angering event, which may have caused participants to focus differently on the object of their anger, and confounded the broadening manipulation. Consequently, Study 7 used more neutral stimuli to manipulate cognitive scope.

### Methods

#### Participants

An *a priori* power analysis indicated that a sample of 88 participants was required to detect an effect size of *d* = 0.50 with 75% power, using a one-tailed criterion of significance. Because of the small number of individuals available in the participant pool, only 87 undergraduate students from the University of New South Wales were recruited. They participated in exchange for partial course credit. Five participants were excluded: one participant for completing a similar task within the past 12 months, and four participants for not following task instructions, leaving 82 participants for analysis (53 females). The final sample had a mean age of 19.59 (*SD* = 4.36), and reported the following ethnicities: White/Caucasian (50%), North East Asian (15.85%), South/Central Asian (8.54%), South East Asian (20.73%), Middle Eastern (3.66%), South American/Central American (1.22%). Participants provided written informed consent.

#### Materials and Procedure

After providing informed consent and demographic information, participants completed the DEQ. They were then randomly assigned to complete a task designed to broaden or narrow cognitive scope. Participants were instructed to provide explanations for ‘how’ (narrow) or ‘why’ (broad) a person was taking an action in four neutrally valenced pictures. Research finds that asking individuals “why” they engaged in an action causes them to focus on global features and activates high level construals, whereas asking an individual “how” they engaged in an action causes them to focus on local features and activates low level construals (Fujita et al., 2006). Thus, asking “why” should broaden cognitive scope by directing attention to increasingly more general details, and asking “how” should narrow cognitive scope by directing attention to increasingly more specific details. Next, participants played a game of Cyberball in which they were excluded as in Peterson and Harmon-Jones (2012). Then, they completed a second cognitive scope task, the Anderson Word Accessibility task, and the DEQ to assess their discrete emotions during the Cyberball task.

### Results and Discussion

Self-reported anger significantly increased in both the broad and narrow conditions after being socially excluded [*t*(41) = 4.68, *p* < 0.001; *t*(41) = 3.96, *p* < 0.001, respectively]. Cognitive scope conditions did not differ significantly on DEQ Anger in response to Cyberball ostracism and the means were not in the expected direction (Table 7). Angry/aggressive word accessibility was marginally significantly greater in the narrow condition, as expected (Table 7).

**Table 7:** Effect of cognitive scope condition on state anger and angry word accessibility – Study 7.

	Narrow (*n* = 41)		Broad (*n* = 41)				
	*M* (*SD*)	95% CI	*M* (*SD*)	95% CI	*t*	*p*	*d*
DEQ Anger	1.80 (0.87)	[1.52, 2.07]	1.96 (0.90)	[1.67, 2.24]	–0.81	*ns*	0.179
AWCT	9.22 (2.81)	[8.33, 10.11]	8.32 (2.81)	[7.43, 9.20]	1.46	0.075	0.276

*AWCT, Anderson Word Completion Task. Because one-tailed *t*-tests were conducted, where means indicate that results were not in the predicted direction, *p*-values are reported as non-significant, abbreviated *ns*.*

## Meta-Analysis

The results of the individual studies provided mixed support for the prediction that increasing cognitive scope would reduce anger. Results of some of the studies showed reductions in anger-related variables following cognitive broadening, and although other studies did not produce results that reached conventional levels of significance, the mean differences were largely in the predicted direction. To assess the reliability of these findings, we conducted a mini meta-analysis using the procedure recommended by Goh et al. (2016).

All anger variables included in the meta-analysis were standardized, and in studies comprising more than one variable of interest, standardized scores were averaged to form a composite anger variable (Table 8). *T*-tests were then conducted to assess the difference in these variables between broad and narrow cognitive scope conditions. For each comparison the *t* score was converted to a *z* score; this *z* score was then used to create a summary weighted *z* score, which was converted to an overall *r* score.

**Table 8:** Study designs and variables included in meta-analysis.

Study	Conditions	IV(s)	Meta-Analysis
1	2 (emotion: anger/neutral) × 2 (cognitive scope: narrow/broad)	Insulting vs. neutral radio broadcast; Sentence unscrambling task	*2 conditions*: narrow vs. broad *DVs*: ATA, Average of AQ subscales, PANAS-X state anger
2	2 (cognitive scope: narrow/broad)	Sentence unscrambling task	*2 conditions*: narrow vs. broad *DVs*: ATA, Average of AQ subscales
3	2 (cognitive scope: narrow/broad)	Sentence unscrambling task	*2 conditions*: narrow vs. broad *DVs*: ATA, Average of AQ subscales
4	2 (cognitive scope: narrow/broad)	Sentence unscrambling task	*2 conditions*: narrow vs. broad *DVs*: ATA, Anger judgements on angry scenes
5	2 (cognitive scope: narrow/broad)	Sentence unscrambling task	*2 conditions*: narrow vs. broad *DVs*: ATA, Anger judgements on angry scenes
6	2 (cognitive scope: narrow/broad)	Elaborate on single (narrow) or different (broad) reasons someone angered them	*2 conditions*: narrow vs. broad *DVs*: Average of AWCT, angry motivation, DEQ anger
7	2 (cognitive scope: narrow/broad)	Explain how (narrow) or why (broad) person was taking an action in neutrally valenced images	*2 conditions*: narrow vs. broad *DVs*: Average of AWCT, DEQ anger

*IV, Independent Variable; DV, Dependent Variable; AQ, Aggression Questionnaire; ATA, Attitudes toward Anger subscale; DEQ, Discrete Emotions Questionnaire; AWCT, Anderson Word Completion Task.*

Attitudes toward anger were measured in Studies 1–5. Results of the meta-analyses indicated that difference in attitudes toward anger between broad and narrow cognitive scope conditions was small in magnitude and statistically significant, overall *r* = 0.100, *p* = 0.018 (Table 9).

**Table 9:** Meta-analysis of effect of cognitive scope condition on attitudes toward anger.

	Narrow	Broad					
	*M* (*SD*)	*M* (*SD*)	*N*	*t*	*d*	*r*	*z*
Study 1	0.16 (1.07)	–0.16 (0.91)	107	1.65	0.322	0.159	0.160
Study 2	0.33 (1.07)	–0.27 (0.87)	51	2.19	0.625	0.298	0.308
Study 3	0.09 (1.09)	–0.09 (0.90)	86	0.86	0.188	0.093	0.094
Study 4	0.19 (1.26)	–0.16 (0.72)	63	1.39	0.356	0.175	0.177
Study 5	–0.07 (0.96)	0.09 (1.05)	116	–0.86	–0.161	–0.080	–0.081

Trait anger/aggression was measured in Studies 1–3. Results of the meta-analysis for this measure were also statistically significant and small in magnitude, overall *r* = 0.127, *p* = 0.021 (see Table 10).

**Table 10:** Meta-analysis of effect of cognitive scope condition on trait anger/aggression.

	Narrow	Broad					
	*M* (*SD*)	*M* (*SD*)	*N*	*t*	*d*	*r*	*z*
Study 1	0.14 (0.77)	–0.15 (0.73)	107	1.98	0.387	0.190	0.192
Study 2	0.24 (0.90)	–0.21 (0.65)	52	2.10	0.593	0.284	0.292
Study 3	–0.04 (0.86)	0.04 (0.84)	86	–0.46	–0.100	–0.050	–0.050

Reported state anger/aggressive motivation was measured in Studies 1, 6, and 7. Results of the meta-analysis for this measure were very small in magnitude and not statistically significant, *r* = -0.035, *p* = 0.657 (Table 11).

**Table 11:** Meta-analysis of effect of cognitive scope condition on state anger/aggressive motivation.

	Narrow	Broad					
	*M* (*SD*)	*M* (*SD*)	*N*	*t*	*d*	*r*	*z*
Study 1	0.04 (1.01)	–0.04 (1.00)	107	0.431	0.084	0.042	0.042
Study 6	–0.07 (0.92)	0.06 (0.77)	98	–0.719	–0.147	–0.073	–0.073
Study 7	–0.09 (0.98)	0.09 (1.02)	82	–0.811	0.181	–0.090	–0.091

Implicit anger/aggression was measured in Studies 4–7. Results of the meta-analysis for this measure were also very small in magnitude and not statistically significant, *r* = 0.040, *p* = 0.130 (Table 12).

**Table 12:** Meta-analysis of effect of cognitive scope condition on implicit anger/aggression.

	Narrow	Broad					
	*M* (*SD*)	*M* (*SD*)	*N*	*t*	*d*	*r*	*z*
Study 4	0.16 (1.03)	–0.13 (0.97)	63	1.14	0.292	0.145	0.146
Study 5	–0.04 (1.02)	0.05 (0.99)	116	–0.48	–0.091	–0.045	–0.045
Study 6	–0.05 (0.94)	0.05 (1.06)	98	–0.47	–0.096	–0.048	–0.048
Study 7	–0.09 (0.99)	0.09 (0.99)	82	–1.46	–0.326	–0.161	–0.162

## General Discussion

Meta-analyses indicated that the overall effect of cognitive broadening on reported attitudes toward anger and trait anger/aggression responses was small in magnitude and statistically significant across the studies. However, meta-analyses did not find a significant overall effect of broadening on reported state anger or implicit anger. Thus, considering evidence from multiple studies combined supports the prediction that broadening cognitive scope reduces some anger-related responses, but that the magnitude of the effect is small.

A large number of exclusions were made in some studies as a result of participants’ difficulty completing the cognitive scope manipulation tasks. This appears to be an unfortunate artifact of the sentence unscrambling manipulation of cognitive scope used in Studies 1-5. However, this task was chosen to be consistent with past studies that have successfully used similar tasks to measure cognitive scope (e.g., Isen and Daubman, 1984; Price and Harmon-Jones, 2010). While participants were better able to complete the conceptually broader manipulations of cognitive scope used in Study 6 and Study 7, these studies did not find support for the prediction that broadening cognitive scope reduces anger responses.

We can only speculate as to the reasons why this program of research found only weak support for the predictions made. Past research consistently found that anger narrows cognitive scope (Gable et al., 2015), and so we predicted that the effect should be reciprocal such that broadening cognitive scope should reduce anger. However, the current results show that manipulating cognitive scope has only a small effect on anger. Perhaps “hot” emotive responses have more influence on “cool” cognitive states than the converse. Or perhaps there is a need for manipulations of cognitive scope to include a self-referential component (e.g., Mischkowski et al., 2012) in order to elicit a stronger effect on anger responses. Alternatively, the manipulation of cognitive scope may not have been effective for some participants. In designing the studies, we considered the inclusion of a manipulation check to test for this. However, we chose not to include one for the following reasons. (1) The ideal placement of the manipulation check would have been immediately following the manipulation but placing it here would have delayed the time between the cognitive scope manipulation and the assessment of anger. We were concerned that this delay would have reduced the effect of the cognitive scope manipulation on anger-related responses. (2) The assessments of the cognitive scope of which we are aware, all take considerable time, and they would further increase the delay between the manipulation and the dependent variables. (3) Inclusion of the manipulation check after the measurement of the dependent variable would likely have led to misleading or inaccurate results, as considerable time had occurred between the manipulation and the assessment of the manipulation check. (4) We thought the manipulation of cognitive scope in most of our studies had high face validity, and that the assessment of a manipulation check was unnecessary. Future research should devise a subtle manipulation check measure that can be included at the appropriate time in the experiment.

The current studies included several diverse measures of angry responses, including self-reported trait anger and aggression, self-reported state anger, self-reported aggressive motivation, implicit anger, and implicit aggression. Although it is possible that some of these measures were particularly insensitive, we take these results to indicate that cognitive scope, as manipulated in the present studies, has only a small effect on angry responses.

It is important to note that individually, these studies were underpowered to detect an effect of the magnitude revealed by the meta-analyses. For example, to detect an effect of *r* = 0.10, the effect for attitudes toward anger which reached statistical significance in the meta-analysis, a sample size of 616 would be required to achieve 0.8 power. To detect an effect of *r* = 0.13, the effect for trait anger and aggression (also significant), a sample of 364 would be required to achieve 0.8 power. Although each of the current studies was under-powered, Braver et al. (2014) propose that in the absence of high levels of statistical power, continuously cumulative meta-analyses, such as we have done, provide an appropriate alternative criterion through which to evaluate the robustness of a phenomenon. Thus, although individually the studies cannot provide conclusive evidence one way or another, when combined they suggest a small effect is present for certain measures of angry responses.

For each study, we reported how we determined sample size. We followed the recommendations of others (e.g., Simmons et al., 2011) who have suggested that when power analyses are not conducted in advance, researchers should report their data-stopping rule. The data stopping rule used in Studies 1, 2, and 4 was that data were collected until the participant pool closed or our allotment has been used. Studies 3, 5, 6, and 7 used power analyses based on a predicted effect size. These studies were conducted almost simultaneously and therefore effect sizes obtained in one study were not used to determine power in another study. In order for a power analysis to be accurate, one needs to know the size of the effect. However, because the question of whether cognitive broadening reduces anger was a novel line of research, it was difficult to know what effect size to predict in advance. The current results revealed that the effect sizes were heterogeneous among the various studies. This is not surprising, because the conceptual variables were operationalized in different ways. Some of the manipulations may have been stronger than others, and some of the measures may have been more sensitive. Additionally, the different populations that were sampled across the studies may have responded differently. After completing all the studies and conducting the mini meta-analysis, we were able to determine that, overall, there is a small effect of broadening reducing anger, but it was not possible to know this prior to conducting and analyzing the studies.

The effect size is small. But it is important to note that the “cause” size is also small. That is, in the current studies, participants were exposed to brief manipulations of a few minutes to induce a broad or narrow cognitive scope, and then their anger-related responses were measured. Had these manipulations (“the cause size”) been conducted over several hours or days, the effect size may have been much larger.

Although the seven studies individually did not find consistent support for the prediction that cognitive broadening can reduce anger, meta-analyses of these studies found a small and statistically significant effect of cognitive broadening on attitudes toward anger and trait anger/aggression. Considering any one of the studies reported in isolation may be misleading. Thus, utilizing meta-analyses on a smaller scale, such as within individual programs of research, is a useful tool to help researchers more accurately take into account new evidence as it emerges (Braver et al., 2014). It is our hope that publishing the findings from this research program will help to inform other researchers in this field, so that they may tailor their future research directions accordingly.

## Data Availability Statement

The raw data supporting the conclusions of this manuscript will be made available by the authors, without undue reservation, to any qualified researcher.

## Ethics Statement

The research was carried out in accordance with the recommendations of Australia’s National Statement on Ethical Conduct in Human Research (for studies conducted by Australian researchers) or the U.S. Department of Health and Human Services, Office of Human Research Protections (for studies conducted by United States researchers) with informed consent from all subjects. All subjects gave informed consent in accordance with the Declaration of Helsinki. The protocol for Studies 1–2 was approved by the Texas A&M University IRB/HSPP. The protocol for Studies 3-7 was approved by the UNSW Human Research Ethics Advisory Panel C: Psychology.

## Author Contributions

ES contributed to the conceptualization and design of the work, data collection, data analysis, writing of the manuscript, and final approval. CH-J and EH-J contributed to the conceptualization and design, data analysis, writing, and final approval. NK, CP, and KK-B contributed to the conceptualization, data collection, writing, and final approval.

## Conflict of Interest Statement

The authors declare that the research was conducted in the absence of any commercial or financial relationships that could be construed as a potential conflict of interest.
