# Supplementary material for: Does Cognitive Broadening Reduce Anger?
Source: Front Psychol. 2019 Jan 8;9:2665. doi: 10.3389/fpsyg.2018.02665 (PMC6332929; doi:10.3389/fpsyg.2018.02665)
Supplement: Supplementary file 1 [file Data_Sheet_1.PDF]

Supplementary Material

Does cognitive broadening reduce anger?

Elizabeth Summerell, Cindy Harmon-Jones, Nicholas J. Kelley, Carly K. Peterson, Klimentina Krstanoska-Blazeska, Eddie Harmon-Jones*

*** Correspondence:** Eddie Harmon-Jones: eddiehj@gmail.com

1 Additional measures and analyses

Some additional measures were completed by participants. Because these questionnaires are not relevant to the current research question, their results have not been analysed or reported in this manuscript. In addition, we include some analyses of other measures that are not relevant to the current research question.

Study 1

In Study 1, participants completed also the Behavioral Inhibition System and Behavioral Activation System scales (BIS/BAS; Carver & White, 1994), the Subjective Happiness Scale (Lyubomirsky, & Lepper, 1999), the Disgust Scale (Haidt, McCauley, & Rozin, 1994), Affect Intensity Measure (Larsen & Diener, 1987), and the 50-item inventory of Goldberg's (1992) Big-Five Factor Markers from the International Personality Item Pool (Goldberg, n.d.).

Study 2

In Study 2, following the cognitive scope manipulation, participants completed the Differential Emotions Scale (DES; Izard, Libero, Putnam, & Haynes, 1993), the Attributional Complexity scale (Fletcher, Danilovics, Fernandez, Peterson, & Reeder, 1986) and items for a new scale that is being developed.

Study 3

In Study 3, prior to the cognitive scope manipulation, participants completed exploratory items for a trait emotion measure that was being developed as part of a different line of research.

Study 4

In Study 4, prior to the cognitive scope manipulation, participants completed the DES. DES Anger was significantly higher in the narrow (M = 2.65, SD = 0.75, 95% CI [2.37, 2.94]) compared to the broad (M = 2.29, SD = 0.62, 95% CI [2.07, 2.50]) cognitive scope condition, $t(61)$ =2.14, p = .036, d = .538. For consistency of analyses, this was not included as a covariate.

In addition, determination and joy scenes were included. Participants in the broad condition (M = 6.94, SD = 0.85, 95% CI [6.65, 7.24]) rated determination scenes as significantly less determined than those in the narrow condition (M = 7.43, SD = 0.95, 95% CI [7.06, 7.80]), $t(61)$ = 2.13, p = .037 (two-tailed),

d = .538. The observed reduction in determination scores is consistent with past research showing that cognitive broadening reduces motivationally intense emotions (Gable & Harmon-Jones, 2011b; 2012). Ratings of enjoyment scenes did not differ between participants in the broad (*M* = 6.01, *SD* = 1.30, 95% CI [5.56, 6.46]) and narrow (*M* = 6.12, *SD* = 1.26, 95% CI = [5.63, 6.61]) cognitive scope conditions *t* = 0.34, *p* = .735 (two-tailed).

Because a high percentage of participants had difficulty following the cognitive scope instructions, exploratory analyses were conducted excluding participants for whom English was a second language. Ratings for determination scenes significantly differed between cognitive scope conditions, *t*(47) = 3.42, *p* = .001 (two-tailed). Participants in the narrow condition (*M* = 7.68, *SD* = 0.78, 95% CI [7.33, 8.04]) reported higher ratings of determination, relative to the broad condition (*M* = 6.87, *SD* = 0.86, 95% CI [6.54, 7.20]). There were no differences between conditions for anger or enjoyment ratings [*t*(47) = 1.68, *p* = .10 (two-tailed); *t*(47) = 0.10, *p* = .92 (two-tailed), respectively]. This suggests that ease of processing the English language may be critical for the cognitive scope manipulation to be successful.

Study 5

In Study 5, prior to the cognitive scope manipulation, participants completed the DES. DES Anger did not differ significantly between the narrow (*M* = 2.18, *SD* = 0.74, 95% CI [1.99, 2.37]) and broad (*M* = 2.32, *SD* = 0.82, 95% CI [2.10, 2.55]) cognitive scope conditions, *t*(114) = 0.97, *p* = .333, *d* = .180.

Determination and enjoyment scenes were also included. Ratings of determination scenes did not significantly differ between participants in the broad (*M* = 7.60, *SD* = 1.01, 95% CI [7.32, 7.88]) and narrow (*M* = 7.29, *SD* = 1.00, 95% CI [7.04, 7.54]) cognitive scope conditions, *t*(114) = 1.66, *p* = .100 (two-tailed). Ratings of enjoyment scenes did not significantly differ between broad (*M* = 6.54, *SD* = 1.23, 95% CI [6.20, 6.88]) and narrow (*M* = 6.44, *SD* = 1.05, 95% CI [6.18, 6.71]) cognitive scope conditions, *t*(114) = 0.44, *p* = .661 (two-tailed).

Study 6

In Study 6, participants completed the Discrete Emotions Questionnaire (DEQ; Harmon-Jones et al., 2016) at the beginning of the study, to rate their emotional responses at baseline. No significant difference occurred in baseline anger between participants in the broad (*M* = 1.36, *SD* = 1.07, 95% CI [1.06, 1.66]) or narrow (*M* = 1.42, *SD* = 1.11, 95% CI [1.09, 1.75]) cognitive scope conditions, *t*(96) = 0.26, *p* = .794 (two-tailed).

Study 7

In Study 7, participants completed the DEQ at the beginning of the study, to rate their emotional responses at baseline. No significant difference occurred in baseline anger between participants in the broad (*M* = 1.21, *SD* = 0.49, 95% CI [1.05, 1.36]) and narrow (*M* = 1.16, *SD* = 0.56, 95% CI [0.98, 1.34]) cognitive scope conditions *t*(80) = 0.42, *p* = .677 (two-tailed).

Finally, participants answered manipulation check questions detailing how included or rejected they felt during the Cyberball task, and were asked to estimate what percentage of the time the ball was thrown to them.

References to citations only mentioned in these supplementary materials

Carver, C. S., & White, T. L. (1994). Behavioral inhibition, behavioral activation, and affective responses to impending reward and punishment: the BIS/BAS scales. *Journal of Personality and Social Psychology*, *67*(2), 319-333. https://doi.org/10.1037//0022-3514.67.2.319

Fletcher, G. J., Danilovics, P., Fernandez, G., Peterson, D., & Reeder, G. D. (1986). Attributional complexity: An individual differences measure. *Journal of Personality and Social Psychology, 51*(4), 875-884. https://doi.org/10.1037/0022-3514.51.4.875

Haidt, J., McCauley, C., & Rozin, P. (1994). Individual differences in sensitivity to disgust: A scale sampling seven domains of disgust elicitors. *Personality and Individual Differences*, *16*(5), 701-713. https://doi.org/10.1016/0191-8869(94)90212-7

Goldberg, L. R. (n.d.). *Big-Five Factor Markers*. Retrieved from https://ipip.ori.org/newBigFive5broadKey.htm

Goldberg, L. R. (1992). The development of markers for the Big-Five factor structure. *Psychological Assessment*, *4*, 26-42. https://doi.org/10.1037//1040-3590.4.1.26

Izard, C. E., Libero, D. Z., Putnam, P., & Haynes, O. M. (1993). Stability of Emotion Experiences and Their Relations to Traits of Personality. *Journal of Personality and Social Psychology, 64*(5), 847-860. https://doi.org/10.1037/0022-3514.64.5.847

Larsen, R. J., & Diener, E. (1987). Affect intensity as an individual difference characteristic: A review. *Journal of Research in Personality*, *21*(1), 1-39. https://doi.org/10.1016/0092-6566(87)90023-7

Lyubomirsky, S., & Lepper, H. (1999). A measure of subjective happiness: Preliminary reliability and construct validation. *Social Indicators Research, 46*, 137-155 https://doi.org/10.1023/a:1006824100041
